# Oral docetaxel plus encequidar – A pharmacokinetic model and evaluation against IV docetaxel

**DOI:** 10.1007/s10928-024-09913-y

**Published:** 2024-03-19

**Authors:** David Wang, Chris Jackson, Noelyn Hung, Tak Hung, Rudolf Kwan, Wing-Kai Chan, Albert Qin, Natalie J. Hughes-Medlicott, Paul Glue, Stephen Duffull

**Affiliations:** 1 Department of Anaesthesia, Waikato Hospital, Hamilton, New Zealand; 2 Department of Medicine, University of Otago, Dunedin, New Zealand; 3 Department of Pathology, University of Otago, Dunedin, New Zealand; 4 Zenith Technology Limited, Dunedin, New Zealand; 5 Athenex Inc, New York, USA; 6 PharmaEssentia Corporation, Taipei, Taiwan; 7 School of Pharmacy, University of Otago, Dunedin, New Zealand; 8 Department of Psychological Medicine, University of Otago, Dunedin, New Zealand; 9 Certara, Princeton, NJ USA

**Keywords:** Docetaxel, Model informed drug development, Project Optimus

## Abstract

The development of optimized dosing regimens plays a crucial role in oncology drug development. This study focused on the population pharmacokinetic modelling and simulation of docetaxel, comparing the pharmacokinetic exposure of oral docetaxel plus encequidar (oDox + E) with the standard of care intravenous (IV) docetaxel regimen. The aim was to evaluate the feasibility of oDox + E as a potential alternative to IV docetaxel. The article demonstrates an approach which aligns with the FDA’s Project Optimus which aims to improve oncology drug development through model informed drug development (MIDD). The key question answered by this study was whether a feasible regimen of oDox + E existed. The purpose of this question was to provide an early GO / NO-GO decision point to guide drug development and improve development efficiency. Methods: A stepwise approach was employed to develop a population pharmacokinetic model for total and unbound docetaxel plasma concentrations after IV docetaxel and oDox + E administration. Simulations were performed from the final model to assess the probability of target attainment (PTA) for different oDox + E dose regimens (including multiple dose regimens) in relation to IV docetaxel using AUC over effective concentration (AUCOEC) metric across a range of effective concentrations (EC). A Go / No-Go framework was defined—the first part of the framework assessed whether a feasible oDox + E regimen existed (i.e., a PTA ≥ 80%), and the second part defined the conditions to proceed with a Go decision. Results: The overall population pharmacokinetic model consisted of a 3-compartment model with linear elimination, constant bioavailability, constant binding mechanics, and a combined error model. Simulations revealed that single dose oDox + E regimens did not achieve a PTA greater than 80%. However, two- and three-dose regimens at 600 mg achieved PTAs exceeding 80% for certain EC levels. Conclusion: The study demonstrates the benefits of MIDD using oDox + E as a motivating example. A population pharmacokinetic model was developed for the total and unbound concentration in plasma of docetaxel after administration of IV docetaxel and oDox + E. The model was used to simulate oDox + E dose regimens which were compared to the current standard of care IV docetaxel regimen. A GO / NO-GO framework was applied to determine whether oDox + E should progress to the next phase of drug development and whether any conditions should apply. A two or three-dose regimen of oDox + E at 600 mg was able to achieve non-inferior pharmacokinetic exposure to current standard of care IV docetaxel in simulations. A Conditional GO decision was made based on this result and further quantification of the “effective concentration” would improve the ability to optimise the dose regimen.

**Supplementary Information:**

The online version contains supplementary material available at 10.1007/s10928-024-09913-y.

## Introduction

The current paradigm of dose selection in oncology drug development is orientated towards determination of maximum tolerated dose (MTD) in early phase trials. However, this approach often leads to doses and schedules of molecularly targeted therapies that are inadequately characterized before initiating registration trials [1]. Poorly characterized dose and dosing schedule may lead to selection of a dose that provides more toxicity without additional efficacy, severe toxicities that require a high rate of dose reductions, intolerable toxicities that lead to premature discontinuation and missed opportunity for continued benefit from the drug, and potentially persistent or irreversible toxicities that limit the options for receiving benefit of subsequent therapies [2]. To address these issues, the FDA through the Oncology Centre of Excellence (OCE) has launched an initiative known as Project Optimus [3], which aims to reform the dose optimization and selection paradigm in oncology drug development. The focus of Project Optimus is to shift expectations and practice towards use of dose-finding and dose optimisation strategies through use of non-clinical and clinical data acquired at the earliest stage possible [4].

Docetaxel is an important taxane used in the treatment of a wide range of solid tumours including breast, head and neck, stomach, prostate, and non-small cell lung cancer [5–9]. The current standard of care regimen is limited to the intravenous (IV) route of administration due to an absolute oral bioavailability of < 10%. This has been shown to be, in part, due to the activity of intestinal P-glycoprotein (P-gp) efflux pump [10] and CYP3A4 first-past metabolism [11]. Furthermore, the IV formulation includes polysorbate 80 and ethanol as excipients due to the low aqueous solubility of docetaxel. Polysorbate 80, while necessary for the IV formulation, is not completely inert and is associated with an increased risk of hypersensitivity systemic reactions requiring pre-medication with steroids [12]. Binding of docetaxel after IV administration is high, primarily to plasma proteins such as α1-acid glycoprotein (AAG) and the excipient polysorbate 80 which is not present in the oral formulation [13]. The concentration of AAG is variable between cancer patients and has been shown to be a significant predictor of bound exposure [14]. Within the same patient, protein binding to AAG is the same for IV and oral routes of administration. However, the IV formulation has additional binding to polysorbate 80. Therefore, quantification of unbound docetaxel is important for direct comparisons between IV docetaxel and oral formulations.

An oral formulation of docetaxel would confer significant benefits for patients including avoidance of hypersensitivity reactions to polysorbate 80 and the associated steroid premedication [12], and removing the costs, including day stay, and inconvenience of IV access [15]. For the healthcare system, the benefits of oral docetaxel would significantly reduce the resources required to deliver the care for the increasing number of oncology patients [16].

Oral docetaxel with encequidar (oDox + E) is a combination regimen including oral docetaxel (as tablets) given 1 h after 15 mg Encequidar. Encequidar is a novel intestine specific P-gp inhibitor with very little systemic uptake and minimal side effects [17]. Encequidar has been shown to successfully increase the absolute bioavailability of oral paclitaxel, and the combination regimen of oral paclitaxel and encequidar (oDox + P) has progressed to a phase III pre-registration trial [18].

Population pharmacokinetics (popPK) models have been developed for the total concentration of docetaxel after IV docetaxel administration [19, 20]. However, to our knowledge there have been no popPK models published that includes both IV and Oral docetaxel, total and unbound concentrations.

Aligning with Project Optimus, we use a single dose oDox + E phase-1 clinical trial as a motivating example to demonstrate the benefits of implementing MIDD at an early proof of concept phase of drug development prior to further investment in multiple-dose dose-optimisation, bioequivalence, formulation, and covariate studies. In this context, we report the development of a popPK model for the total and unbound plasma docetaxel concentration after both IV docetaxel and oDox + E administration. Simulations from this model is applied to inform a GO / NO-GO decision that aims to determine whether a feasible regimen of oDox + E exists. If no feasible regimen exists, a clear NO-GO can allow early termination of development and investment of resources elsewhere, a clear GO improves confidence in the probability of success at the next phase of development, and a conditional GO would highlight the assumptions that need to be further quantified. The utilisation of MIDD in this case improves the efficiency of the drug development process by facilitating the next step in dose optimisation.

## Methods

The methods describe the processes used to determine whether a feasible oDox + E regimen exists. The methods begin with an outline of the phase I study and data collected from this study. Subsequently, the methods are divided into two parts. Part 1 outlines the pharmacokinetic model development from the data collected and Part 2 outlines the application of the pharmacokinetic model in a GO / NO-GO decision framework to determine future dose optimisation and drug development process.

### Study design

A phase I Open-label, two-study period, two-treatment trial was undertaken and reported previously [21]. The details are briefly described here. Nine patients with metastatic prostate cancer (mPC) undergoing treatment with IV docetaxel were recruited and successfully completed the study. Each patient underwent two phases within the study. The first phase consisted of standard of care IV docetaxel [22] (~ 75 mg/m^2^) infused over 1 h along with premedication prescribed by the treating oncologist. The second phase, at least 3 weeks later, consisted of single dose oDox + E (15 mg encequidar given 1 h prior to oral docetaxel at a dose level of 75 mg/m^2^, 150 mg/m^2^ or 300 mg/m^2^). The safety and tolerability of oDox + E was demonstrated in the phase I trial as no serious adverse effects occurred at any dose level [21].

### Data

**Fig. 1:**
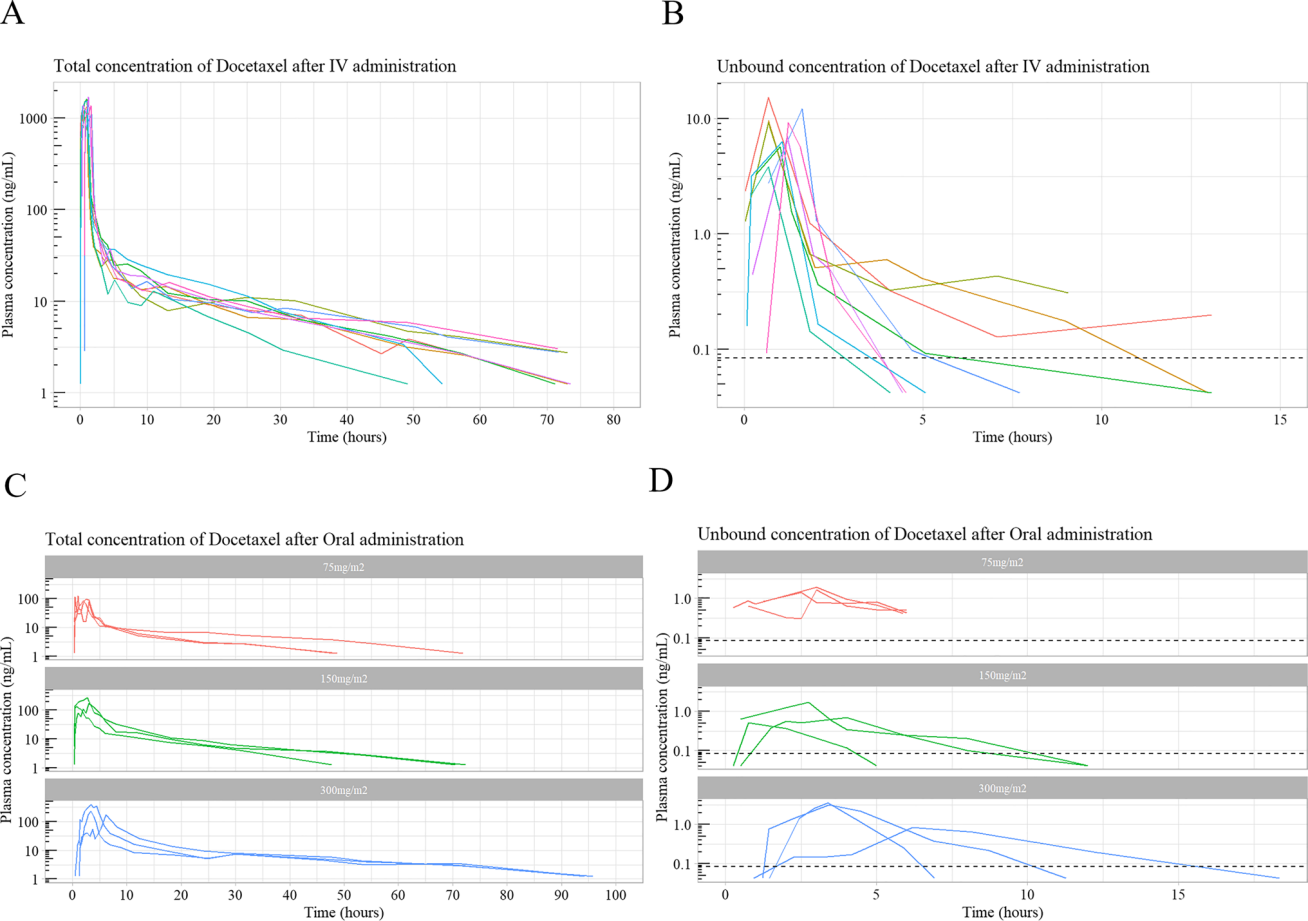
Individuals profiles of total and unbound plasma concentration after administration of IV Docetaxel or oDox + E. Sub-figure A shows the total concentration of docetaxel in plasma after IV docetaxel infusion. Sub-figure B denotes the unbound concentration of docetaxel in plasma after IV docetaxel, dashed line denotes the lower limit of quantification (0.084 ng/mL). Sub-figure C denotes the total concentration of docetaxel in plasma after oral oDox + E for the three dose levels (75 mg/m2, 150 mg/m2 and 300 mg/m2). Sub-figure D denotes the unbound concentration of Docetaxel in plasma after oDox + E at each dose level, dashed line denotes the lower limit of quantification (0.084 ng/mL)

Intensive pharmacokinetic (PK) sampling was undertaken after IV docetaxel and oDox + E. Twenty-four blood samples were taken after IV docetaxel commencement at 0, 2, 5, 8, 12, 20, 40, 60 min, 1.25, 1.5, 1.75, 2, 3, 4, 5, 7, 9, 13, 19, 25, 33, 49, 57, 73 h. 23 blood samples were taken after oDox + E administration at 0, 0.25, 0.5, 0.75, 1, 1.25, 1.5, 2, 2.5, 3, 3.5, 4, 5, 6, 8, 12, 18, 24, 32, 48, 56, 72 and 96 h. Blood samples were immediately centrifuged, and the resultant plasma frozen at -80 °C. Total docetaxel plasma concentrations were measured for all samples using a validated high performance liquid chromatography-mass spectrometry (HPLC-MSMS) assay. Unbound docetaxel plasma concentrations were measured for selected samples using a validated ultrafiltration HPLC-MSMS assay. Selection of samples for unbound analysis was based on optimal design, in total 7 and 8 plasma samples were analysed for unbound docetaxel concentration after IV docetaxel and oDox + E, respectively. Unbound analysis was carried out on a subset of the available plasma samples due to resource constraints (time and capital associated with the unbound assay), optimal design was employed to select the unbound analysis to maximise the information available from a modelling perspective described in our previous manuscript [23]. A total of 135 blood samples were selected for unbound assay and subsequent model-based analysis with 25% below the limit of quantification. Figure 1 shows the spaghetti plots for the plasma concentrations outlined above.

## Methods Part 1 – Pharmacokinetic model development

### Model building

The analysis was performed in Pirana v2.9.7 with Perl speaks NONMEM (PsN) v4.4.8 and NONMEM v7.4.3 using the first-order conditional estimation method with interaction. Between-subject variability (BSV) was implemented using exponential models. A combined residual error model with additive and proportional elements was used for all models. The M6 method [24] was utilised for samples below the limit of quantification (0.084 ng/mL for unbound docetaxel, 2 ng/mL for total docetaxel).

Sub-models were developed sequentially with subsets of the data available as shown in Table 1. Each sub-model was used to explore a model structure with only the relevant data required. Successful model structures were carried forward through to the final model. Covariates were not considered in the model building process due to the limited number of patients. Model feature selection was based on 1) statistical significance, where a decrease in the objective function value of more than 3.84 units (for 1 degree of freedom) corresponds to a P-value < 0.05 (based on the chi-squared distribution with the number of additional parameters representing the degrees of freedom), 2) model stability, e.g., the ability for the model to converge successfully, 3) biological plausibility, i.e. the estimated parameters were externally consistent with other published literature, and 4) clinical significance, i.e. inclusion of a model feature such as time dependent bioavailability would be expected to significantly impact the PK profile, for example, an increase in total systemic exposure of > 20%. Since the 2 observations (total and unbound) were available from each blood sample a statistical model was considered that allowed for correlation between observations from the same blood sample. This, using NONMEM notation, is termed a level 2 statistical model for correlations in the data (hereafter termed “L2”).

**Table 1:** Iterative model building process. Each individual feature of the final model was explored using the minimal amount of data at each sub-model

Sub-model	Structure	Data used	Structural model exploration
1	Structural model for IV docetaxel	**Total** docetaxel concentration profiles after **IV docetaxel.** only	1 Number of compartments for IV docetaxel (1,2 or 3)
2	Structural model for oDox + E	**Total** docetaxel concentration profiles after **oDox + E** only	2.1 Number of compartments for oDox + E (1,2 or 3) 2.2 Lag-time (Lag-time or no lag time)
3	Absorption phase	**Total** docetaxel concentration profiles after **IV docetaxel** and **oDox + E** combined	3 Bioavailability (Constant vs time-varying)
4	Plasma binding for IV docetaxel	**Total** and **unbound** docetaxel concentration profiles after **IV docetaxel**	4 Binding mechanics (Constant, Time-varying, Michaelis–Menten, 2-site binding)
5	Plasma binding for oDox + E	**Total** and **unbound** docetaxel concentration profiles after **oDox + E**	5 Binding mechanics (Constant, Time-varying, Michaelis–Menten, 2-site binding)
Final Model	Residual error correlation	**Total** and **unbound** docetaxel concentration profiles after **IV docetaxel** and **oDox + E**	6 Correlation across errors (L2 or no L2 data item)

The first and second steps of the model building processes explored the structural model for IV docetaxel and oDox + E, respectively (Sub-models 1 and 2 in Table 1). The evaluation was carried out separately using total docetaxel concentration profiles after IV docetaxel or oDox + E administration. The key features evaluated were the number of compartments that best described the total concentration profile (1-, 2- or 3-compartments) and the significance of a lag-time post oDox + E administration.

Next, the extent of absorption of oDox + E was explored (Sub-model 3 in Table 1). The parameter in question was the absolute bioavailability (F) of oDox + E. F was explored using the total docetaxel concentration profiles after both IV docetaxel and oDox + E administration. Constant F and time-varying F were evaluated.

In sub-models 4 and 5, the binding characteristics were explored for IV docetaxel and oDox + E separately. This was the first point in the model building process where total concentration and unbound concentration of docetaxel was modelled simultaneously. Models with constant binding, time-varying binding, Michaelis–Menten binding, and 2-site binding were evaluated for each route of administration (Sub-models 4 and 5 in Table 1). Note as the IV formulation exists as a micelle solution, binding could include elements other than plasma proteins and hence protein binding of IV and oral were considered separately.

Finally, as the unbound concentration of docetaxel was ascertained using the same plasma sample as the corresponding total concentration of docetaxel, the last feature evaluated was whether a correlated error structure using an L2 data item in NONMEM would improve the model (Final model in Table 1). This was incorporated into the final model which represented the statistically and clinically important characteristics from sub-models 1–5.

### Evaluation of final model

The final popPK model was evaluated using goodness of fit plots and individual plots of concentration vs time to determine visually whether the model described the pharmacokinetic data from the patients. Visual predictive checks (VPCs) were not performed due to the limited number of patients.

## Methods Part 2 – Application of model for oDox + E GO / NO-GO decision

### Simulation settings

R version 3.5.2 and the PKPDsim package version 1.1.0 were utilised to perform simulations. The final population pharmacokinetic model for docetaxel and associated parameters shown above were coded in R.

The simulation conditions for IV docetaxel and oDox + E is outlined in Table 2. The range of doses chosen for oDox + E reflects the range of doses given in the clinical trial. The effective concentrations of unbound docetaxel of 0.1 ng/mL to 1 ng/mL in 0.1 ng/mL increments were used to evaluate the probability of target attainment. The range of “effective concentrations” was derived based on reported *in-vitro* IC50 values [25–27] and clinical plasma values that correlated with patient outcomes [19]. As the meaning effective concentration is more general, we have retained its use throughout the article to avoid misinterpretation.

**Table 2:** Summary of simulation conditions. IV docetaxel simulation only includes 1 dose as this set the Area Under the Curve Over Effective Concentration (AUCOEC) Target which the oDox + E simulations were evaluated against

	IV docetaxel	oDox + E
Dose (mg)	130 mg (75 mg/m^2^ × 1.7m^2^)	400 to 600 by 50 mg increments
Dosing regimen	Single infusion over 1 h	Single dose or 1 or 2 repeated doses
Effective concentration (ng/mL)	0.1, 0.2, 0.3, 0.4, 0.6, 0.7, 0.8, 0.9, 1.0	
Simulation length	24 hours	
Simulation steps	3 minutes	

A range of doses were assessed due to variability of reported effective concentrations in clinical studies and in vitro studies.

### Defining the target

The Area Under the Curve Over the Effective Concentration (AUCOEC) was used as a metric to compare standard of care IV docetaxel regimen with the range oDox + E doses.

**Fig. 2:**
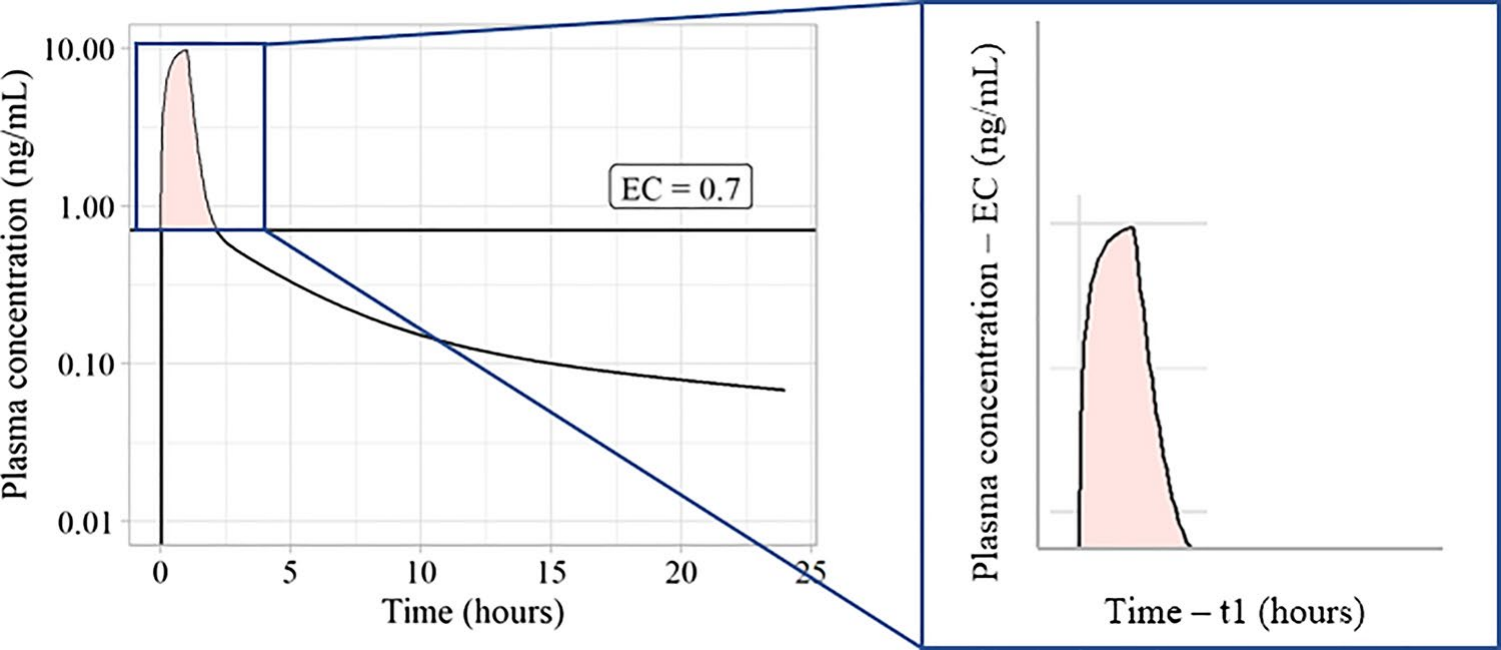
Area under curve over effective concentration (AUCOEC) calculation. The data points below the effective concentration (EC) value were removed and the remaining data points were transformed in the y-axis by subtracting the EC value and in the x-axis by subtracting the first time point which at which the plasma concentration exceeded the EC (t1). The AUC of the remaining data points were calculated which represents the AUCOEC. *Abbreviations: EC – Effective concentration, t1 – First time point at which the data exceeds the EC*

The AUCOEC was calculated as the AUC of the concentration range that was above the EC. This is depicted in Fig. 2.

### Simulations

The population pharmacokinetic model was used to simulate 1000 virtual patients, each with a unique set of pharmacokinetic parameters. From each simulated patient, individual pharmacokinetic profiles were generated for a dose of 130 mg of IV docetaxel over 1 h (the standard of care) and each of the oDox + E doses outlined in Table 2.

### AUCOEC target from IV PK profiles

Each virtual patient was administered the standard of care, IV docetaxel at 130 mg over 1 h and oDox + E at the specified dose levels. Each virtual patient therefore acted as their own control. No between occasion variability, carryover, period, or sequence effects were included in the simulation and each patient received both formulations once only with an assumed washout between dosing occasions.

The AUCOEC-Target was calculated as the AUCOEC of the IV docetaxel regimen at each EC level for each patient minus a 20% non-inferiority margin, i.e., AUCOEC-Target = AUCOEC for the 130 mg IV docetaxel dose – 20%. A 20% non-inferiority margin was utilised based on the accepted bioequivalence error margin [28].

### Probability of target attainment

**Fig. 3:**
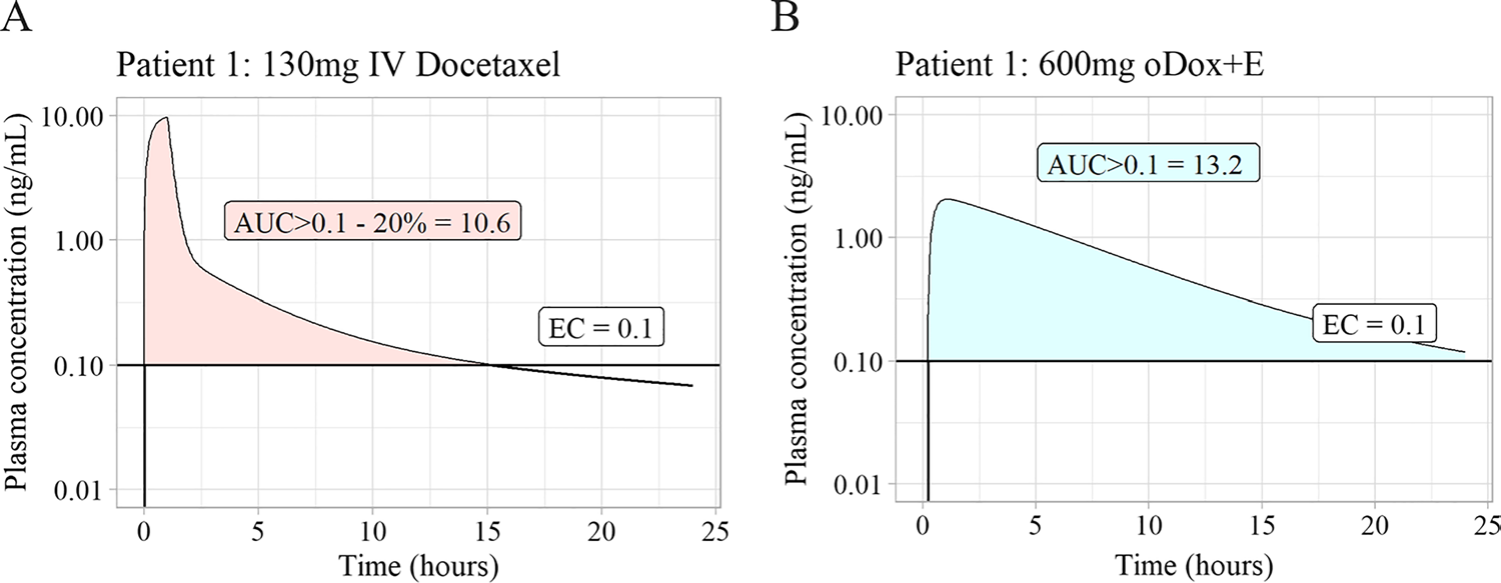
Comparison of AUCOEC of oDox + E with AUCOEC-Target. The left plot shows the AUCOEC for an IV dose of docetaxel for a hypothetical patient, minus 20%. The right plot shows the AUCOEC for an oDox + E dose of 600 mg and EC of 0.1 ng/mL (Cyan) for the same patient which can then be compared to the left plot. The AUCOEC-Target is derived from the AUCOEC for IV docetaxel minus 20%. If the AUCOEC for oDox + E (cyan) is greater than the AUCOEC-Target then a “success” (s = 1) is counted, otherwise a “fail” (s = 0) is counted. In this example, the AUCOEC for oDox + E is 13.2 while the AUCOEC-Target is 10.6 resulting in a “success” (s = 1). Note the y-axis are shown in the log-scale, direct comparison of the areas highlight may be misleading

Each oDox + E PK profile was evaluated against the AUCOEC-Target set by the IV simulations at the corresponding EC level, the comparison resulted in a success (s = 1) or failure (s = 0) and is expressed in Eq.1. A success for an oDox + E PK profile occurred when an oDox + E dose for a particular patient produced an AUCOEC greater than or equal to the AUCOEC-Target set by a 130 mg IV docetaxel regimen given to the same simulated patient. Figure 3 shows the calculation of the AUCOEC for an oDox + E dose of 600 mg at the 0.1 ng/mL EC level and the corresponding AUCOEC-Target, note this comparison is performed for every oDox + E and EC combination across every simulated patient. Finally, the PTA was calculated as the number of oDox + E “successes” at a given dose and EC, divided by 1000. The calculation of PTA is shown in Eq.2.

1 $$\left\{\begin{array}{l}{\Psi }_{oDox+E }\ge {\Psi }_{Standard\, of\, care\, IV\, docetaxel }-\Delta , s=1\\ Otherwise , s=0\end{array}\right.$$

Equation 1 comparison of a single oDox + E simulation against standard of care IV docetaxel within the same simulated patient.

*Where* ${\Psi }_{oDox+E}$ *is AUCOEC of a particular oDox* + *E dose at a specific EC for a single patient with a unique set of pharmacokinetic parameters, *${\Psi }_{Standard of care IV docetaxel}$* is the AUCOEC of 130 mg IV docetaxel infused over 1 h for the same simulated patient at the same EC, *$\Delta$* is the non-inferiority margin set at 20%,* $s$ = *1 denotes a success where the AUCOEC for oDox* + *E is greater than or equal to the target AUCOEC set by IV docetaxel and* $s$ = *0 denotes a failure where the AUCOEC for oDox* + *E is less than the AUCOEC-Target.*

2 $$PTA (\%)= \frac{{\sum }_{n}^{i=1}{s}_{i}}{n} \times 100$$

Equation 2 probability Target Attainment (PTA).

*PTA is probability target attainment (%) for a particular oDox* + *E dose and EC level, *${\sum }_{n}^{i=1}{s}_{i}$* is the count of the number of oDox* + *E simulations with AUCOEC which were equal to or greater than the AUCOEC-Target(i.e., s* = *1), n is the number of simulations (1000).*

**Fig. 4:**
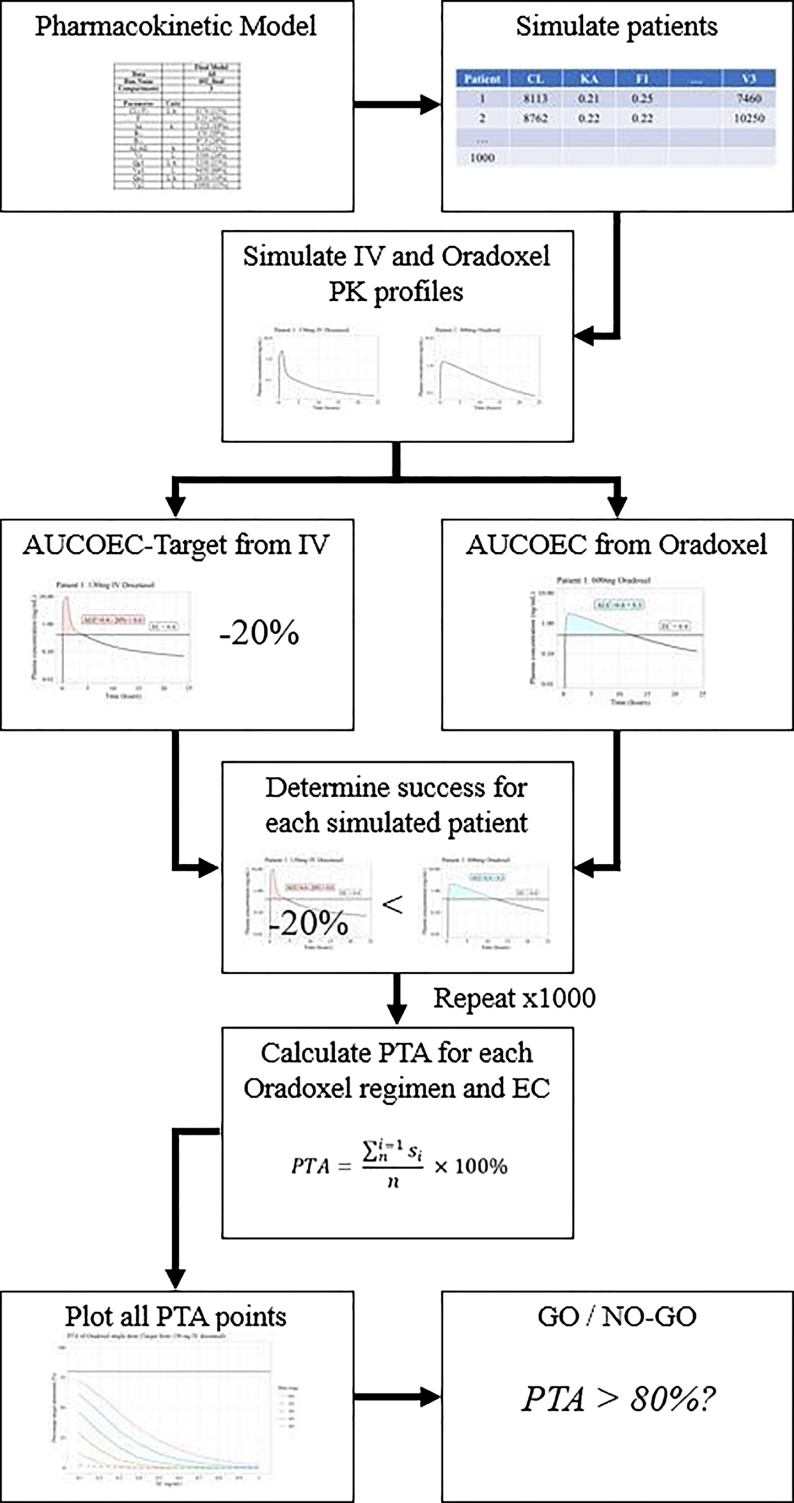
Simulation and GO / NO-GO workflow. Abbreviations: PK – Pharmacokinetics; AUCOEC—Area under curve over effective concentration; PTA – Probability target attainment

Note, the application of the non-inferiority margin in this context is different to that of non-inferiority clinical trials, where the non-inferiority margin is applied to the results of a reference trial and hence includes a confidence band. Here each simulated oDox + E dose regimen is compared to the standard IV docetaxel regimen given to the same patient. The non-inferiority margin is applied to the AUCOEC of the standard IV docetaxel regimen for each patient before being used to determine whether an oDox + E dose regimen was a success or fail. Therefore, the non-inferiority was applied to each simulated patient based on their unique set of pharmacokinetic parameters. Hence, the PTA values calculated for the oDox + E regimens reflects a direct pair-wise comparison with the standard of care IV docetaxel regimen. The overall simulation workflow is shown in Fig. 4.

### Multiple dosing and oral docetaxel

The steps above outlined the calculation of AUCOEC and PTA for a single dose of oDox + E. The AUCOEC and PTA after two and three doses of oDox + E at each of the dose levels between 400 and 600 mg was also calculated. The repeat dose time was selected to be 24 h as unbound docetaxel was essentially undetectable at 12 h for almost all patients after oDox + E. The AUCOEC-Target was determined from a single dose of IV docetaxel. In this situation a complete washout was assumed between consecutive oDox + E doses and hence the total AUC of a multi-dose regimen was the product of the AUC of a single dose and the number of doses in the multi-dose regimen. The PTA was then calculated in the same way with the same AUCOEC-Target.

### Comparison without P-gp inhibitor

A 600 mg dose of oral docetaxel with a bioavailability of 8% [10] given as a single dose, two-doses and three-doses were also simulated and evaluated against the respective AUCOEC-Targets set by the standard of care IV docetaxel regimen for each EC level and was plotted alongside oDox + E PTA values. This allowed comparison of oral docetaxel (without any P-gp inhibition and enhancement of bioavailability) with oDox + E.

### GO / NO-GO decision framework

The GO / NO-GO decision for oDox + E was based on the PTA values obtained from the simulations. The PTA can be interpreted as the probability a dose regimen of oDox + E would be expected to produce the same or greater exposure compared to the standard of care IV docetaxel regimen at a particular EC level. A PTA threshold of 80% was deemed sufficient for an oDox + E dose regimen to be considered non-inferior to the standard of care IV docetaxel regimen. The 80% threshold for PTA can be thought as the power for each simulation. The decision framework consisted of 2 parts:

1. GO / NO-GO decision – existence of a practical regimen of oDox + E

If any oDox + E single, double, or triple dose below or equal to 600 mg per dose resulted in a PTA of > 80% for any EC level then a practical regimen is said to exist, and the GO conditions below applied. If the PTA did not exceed 80% for any dose level and EC combination, then this indicates non-existence of the necessary criteria, and a NO-GO decision would be made.

2. GO conditions.

The existence of a potential oDox + E regimen does not itself imply a success in future development. It is possible in some cases that oDox + E might not attain a PTA > 80% for all values of EC. Therefore, the GO recommendation is complicated by uncertainty around the exact EC level. Hence two scenarios could arise when a practical oDox + E regimen is shown to exist:

- If oDox + E achieved a PTA of greater than 80% at all EC levels, then a clear GO recommendation would be made.
- If oDox + E achieved a PTA of greater than 80% at some EC levels and not others, then a conditional GO recommendation would be made dependent on the confidence in the highest EC level which produces a PTA of greater than 80%. This GO condition would contain the condition that further pharmacological study be required to quantify an EC (or similar metric) in order to assess the probability of success of subsequent Phase II trials.

## Results

### Results part 1 – Pharmacokinetic model development

**Fig. 5:**
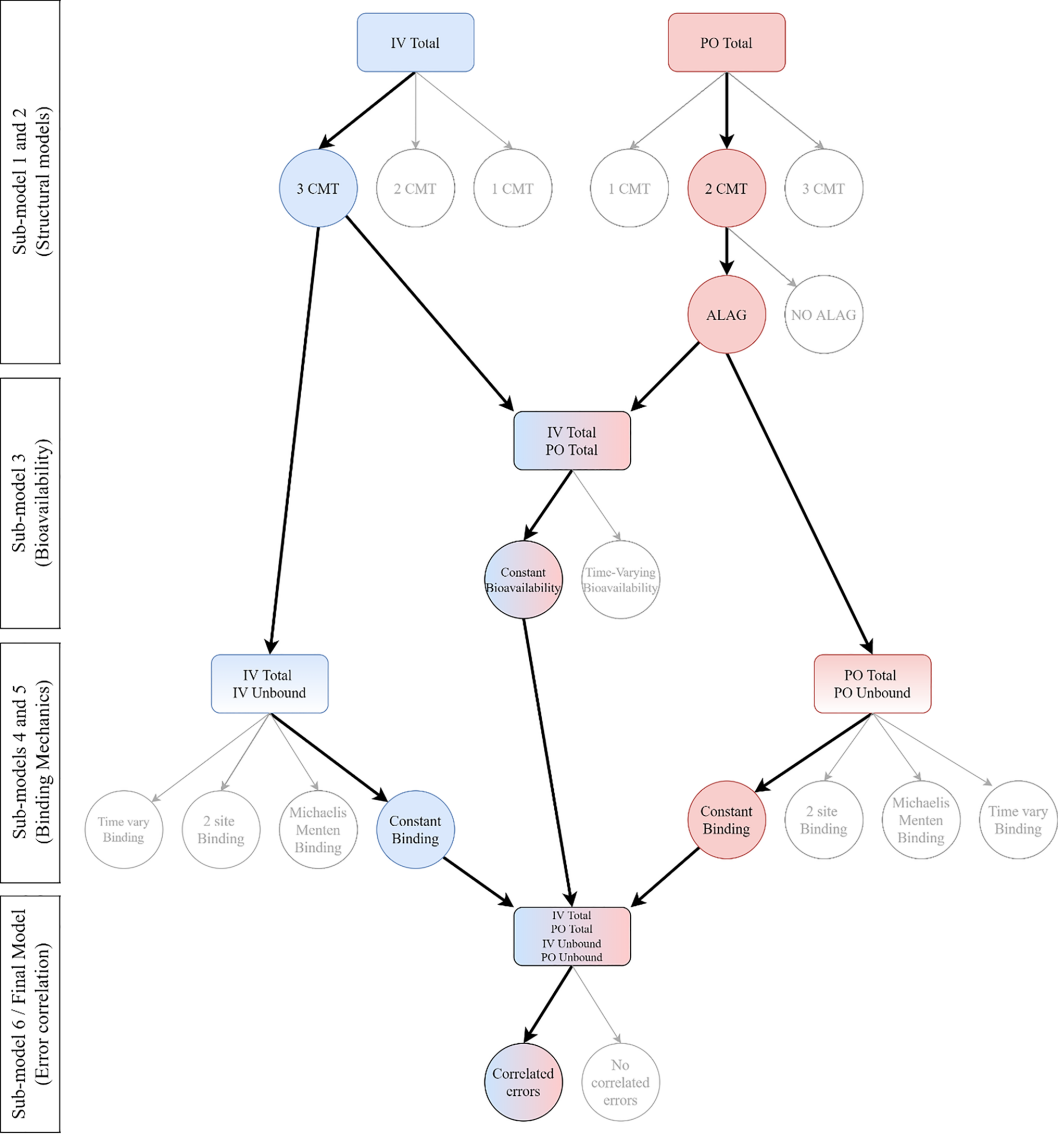
Iterative model building process. The subset of pharmacokinetic data used are shown in the rectangles. For example, “IV Total” denotes the total concentration profiles of docetaxel after intravenous administration. “PO Total | PO unbound” denotes the total concentration and unbound concentration profiles of oDox + E after administration. “IV Total | PO Total | IV Unbound | PO Unbound” denotes the entire dataset. The circles represent the model feature that was evaluated from the dataset, the bold arrows point to a model feature contained in a coloured circle that best described the data while the thin arrows show the model features contained in the grey circles that were evaluated alongside the chosen model feature. The colour blue is used to represent IV data and models while red represents oDox + E data and models. The combination of colours represents both are present*. Abbreviations:IV Total* *– Dataset of total concentration of docetaxel in plasma over time after IV administration of docetaxel; PO Total – Dataset of total concentration of docetaxel in plasma over time after oDox* + *E administration; 1CMT – One compartment structural model; 2CMT – Two compartment structural model; 3CMT – Three compartment structural model; IV Total | IV Unbound – Dataset of total and unbound concentration of docetaxel in plasma over time after IV administration of docetaxel; IV Total | PO Total – Dataset of total concentration of docetaxel in plasma over time after IV administration of docetaxel and oDox* + *E administration; PO Total | PO Unbound – Dataset of total and unbound concentration of docetaxel in plasma over time after oDox* + *E administration; Time vary Binding – Model with time varying binding mechanics of docetaxel to plasma proteins; 2 site Binding – Model with 2 site binding mechanics of docetaxel to plasma proteins; Michaelis Menten Binding – Model with Michaelis Menten binding mechanics of docetaxel to plasma proteins; Constant Binding – Model with constant binding mechanics of docetaxel to plasma proteins. Constant Bioavailability – Model with a constant bioavailability throughout; Time Varying Bioavailability – Model that allows bioavailability to change with time; IV Total | PO Total | IV Unbound | PO Unbound – Entire dataset containing total and unbound concentration of docetaxel in plasma over time after IV administration of docetaxel and oDox* + *E administration; Correlated errors – L2 data item used within the model to assess for correlated errors between unbound and total concentration plasma samples; No correlated errors – L2 data item not used within the model*

The key model building decision points are shown in Fig. 5. Model structures (open and filled circles) were evaluated sequentially based on data (shown in the rectangles). The best structures (shown as filled coloured circles) were carried forward into the next part of model building.

#### Sub-model 1 (IV docetaxel structural model)

A 3-compartment model best described the total plasma concentration of docetaxel after IV docetaxel administration.

#### Sub-model 2 (oDox + E structural model)

A 2-compartment model with lag-time best described the total plasma concentration of docetaxel after oDox + E administration.

#### Sub-model 3 (oDox + E absorption phase)

Time-varying bioavailability was explored in sub-model 3 and did not result in a statistically significant improvement in the objective function; therefore, a constant bioavailability was carried over to subsequent models.

#### Sub-model 4 (Plasma binding for IV docetaxel)

Different binding mechanics of docetaxel after IV docetaxel administration were explored. For IV docetaxel, binding can be to protein or the excipient Polysorbate 80. Time-varying, Michaelis–Menten and 2-site binding mechanisms did not produce statistically significant improvements in the objective function; therefore, a constant binding mechanism (i.e., Unbound concentration equals total concentration multiplied by fraction unbound) was carried over to subsequent models. The parameters for sub-model 4 is shown in Supplement 1 “IV Docetaxel Model” column.

#### Sub-model 5 (Plasma binding for oDox + E)

Different binding mechanics of docetaxel after IV docetaxel administration were explored. For oDox + E, binding is to protein only. Time-varying, Michaelis–Menten and 2-site binding mechanisms did not produce statistically significant improvements in the objective function; therefore, a constant binding mechanism was carried over to subsequent models. The parameters for sub-model 5 is shown in Supplement 1 “oDox + E Model” column.

#### Final model (Sub-model 6)

A 3-compartment model with linear elimination, constant bioavailability, constant binding mechanics, and a combined error model with the L2 data item provided the best fit for the pharmacokinetic data collected which consisted of total and unbound concentrations of docetaxel in plasma after IV docetaxel and oDox + E administration.

The parameters of the final model are shown in Supplement 1. The structure of the final model is shown in Supplement 2.

The parameters in Supplement 1 are reported in the context of unbound docetaxel. The moderate to large standard errors are expected given the dataset size of 9 patients (i.e., proportional to 1/sqrt(n)) and the number of parameters included in the model. Of note, the residual standard errors of Vp1 & ω_CL_ were marginally high, however, this would not invalidate the simulations from the model and the ability to determine whether a feasible regimen exists through the GO / NO-GO framework discussed below remains unchanged.

The parameters can be compared to the total docetaxel pharmacokinetic parameters by an adjustment using the binding constant. For example, the clearance of unbound docetaxel in the final model is estimated at 8570 L h^−1^ with a fraction unbound of 0.67%. Therefore, the total IV docetaxel clearance is 8570 multiplied by 0.67% which is approximately 57.3 L h^−1^.

#### Individual and Goodness of fit plots for final model

**Fig. 6:**
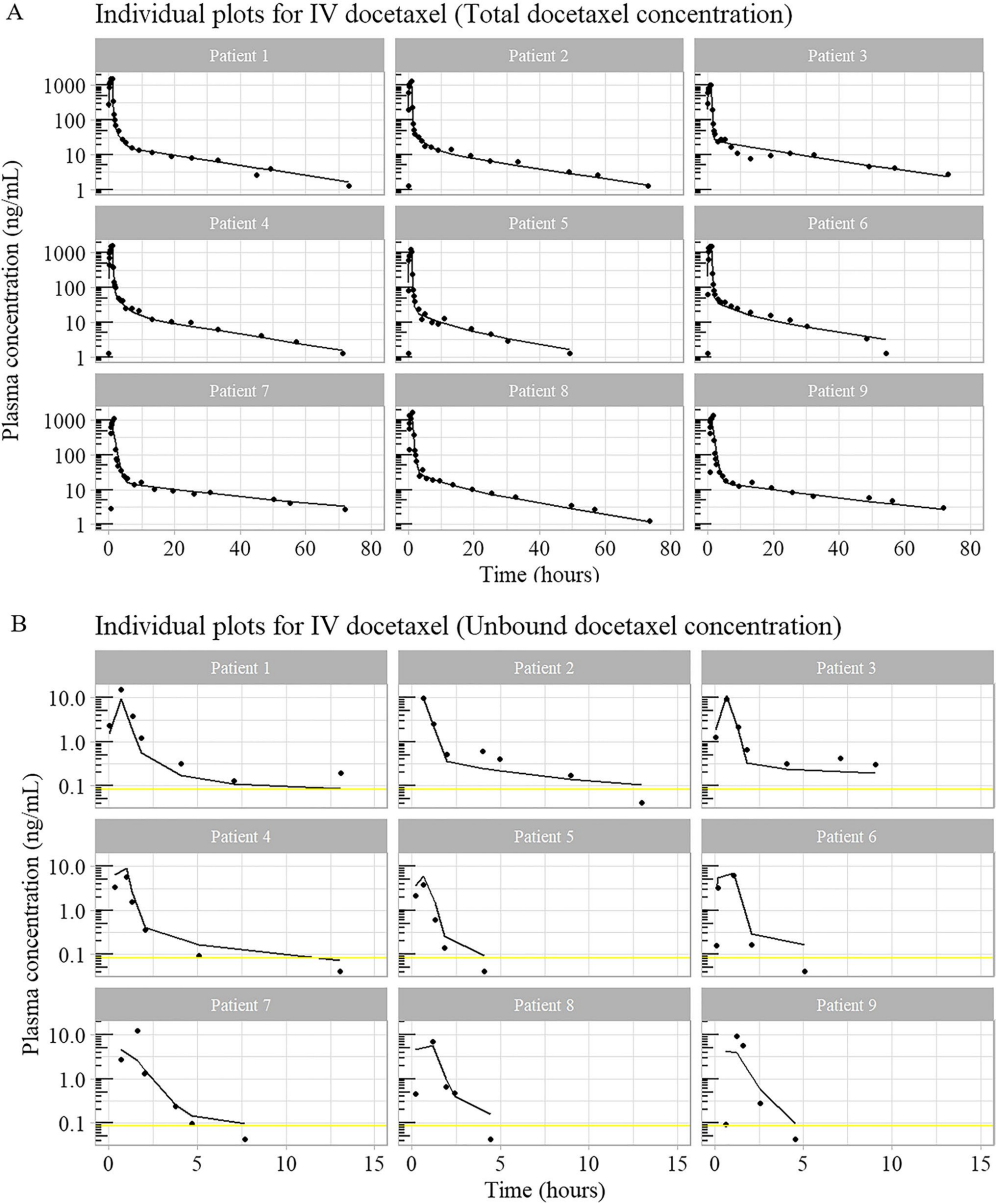

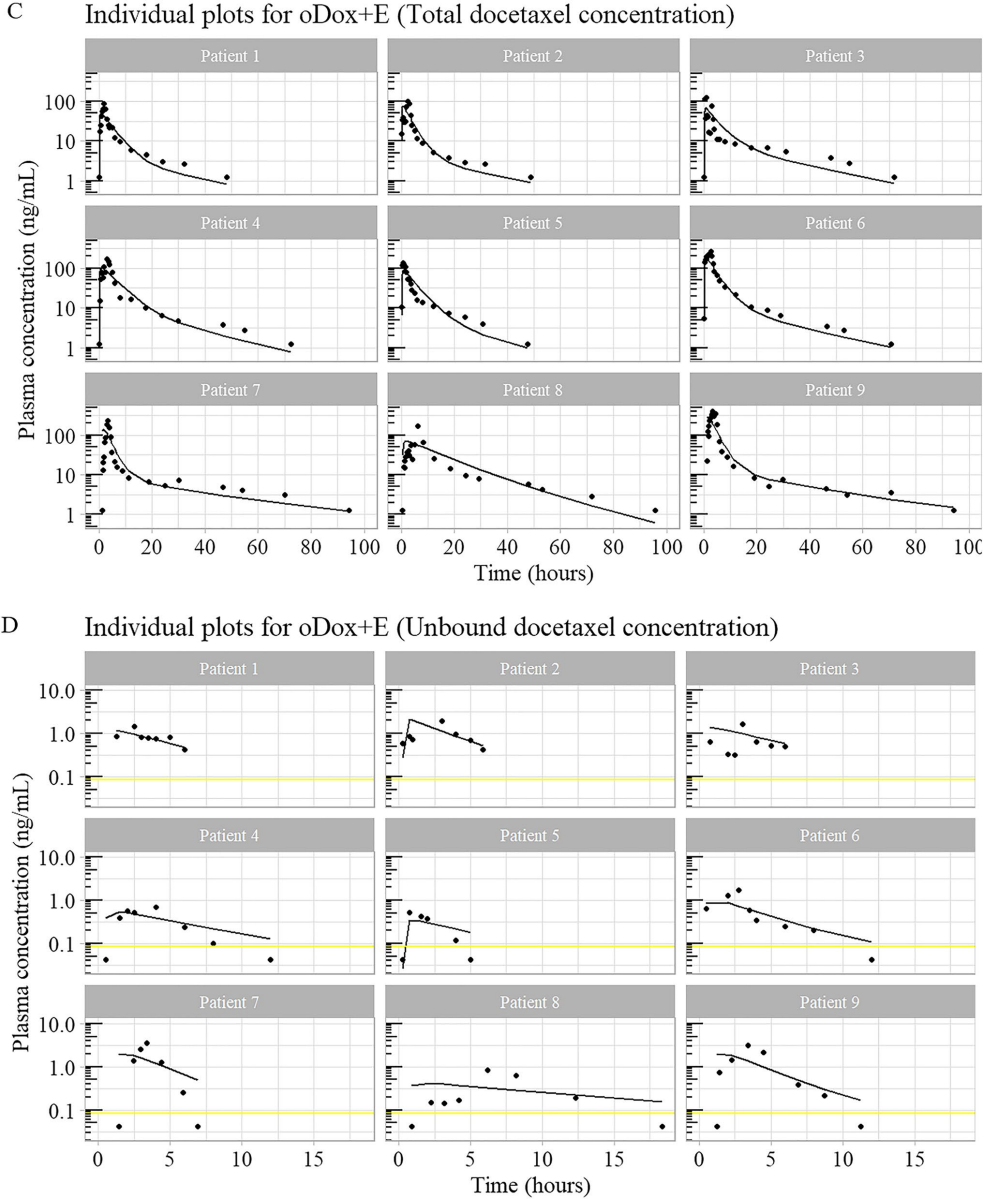
Individual plots of total concentration, unbound concentration of docetaxel after administration of IV docetaxel and oDox + E. The yellow line denotes the lower limit of quantification of 0.084 ng/mL for unbound docetaxel

Figure 6 shows the individual plots for the total concentration and unbound concentration of docetaxel in plasma after IV docetaxel and oDox + E administration for each patient. The goodness of fit plots are shown in Supplement 3 for total docetaxel and unbound docetaxel samples by route of administration.

The individual predictions for the total plasma concentration and unbound plasma concentration of IV docetaxel are shown in Fig. 6A and B, respectively. The predictions closely match the measured plasma concentrations. The individual predictions for the total plasma concentration after oDox + E administration are shown in Fig. 6C and the predictions generally match the measured plasma concentrations, although not as closely when compared to IV docetaxel, suggesting more variability in the oDox + E that has not been explained by the model. The individual predictions for unbound plasma concentration after oDox + E administration are shown in Fig. 6D and appears to only capture the profiles partly, however, this is expected given the very small concentrations that the model is working with for unbound oDox + E (i.e., the peak concentrations are around 1 ng/mL, roughly only 10-times greater that the LLOQ of 0.084 ng/mL). This highlights the importance of modelling the unbound alongside total concentration samples to better estimate the pharmacokinetic model parameters.

The goodness of fit plots for IV docetaxel both bound and unbound is close to linear suggesting an adequate fit (Supplement 3A, right graph and Supplement 3B, right graph). The goodness of fit plot for total concentration after oDox + E has a U-shaped curve which suggests model-misspecification (Supplement 3A, left graph). The goodness of fit for unbound docetaxel after oDox + E (Supplement 3B, left graph) appears to be linear, but this is hard to interpret with confidence due to the small concentrations involved.

### Results part 2 – Application of model for oDox + E GO / NO-GO decision

#### Simulation results

**Fig. 7:**
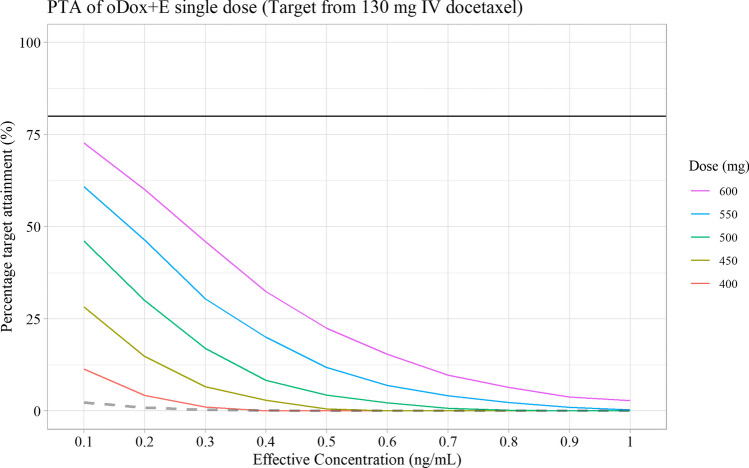
Probability target attainment of oDox + E single dose regimens between 400 to 600 mg by dose level. Black line denotes 80% PTA. Dashed line denotes the PTA of a 600 mg dose of oral docetaxel alone (i.e., bioavailability of 8%). PTA – Probability target attainment

**Fig. 8:**
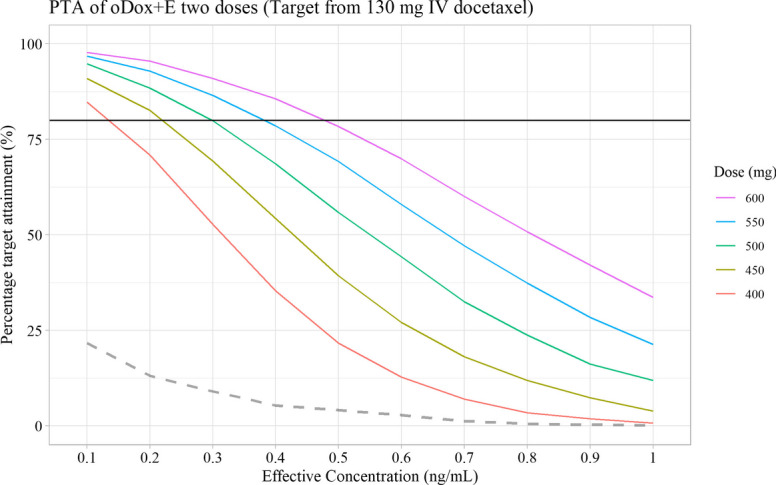
Probability target attainment of oDox + E dose between 400 to 600 mg given twice by dose level. Black line denotes 80% PTA. Dashed line denotes the PTA of a 600 mg dose of oral docetaxel alone (i.e., bioavailability of 8%) given twice. PTA – Probability target attainment

**Fig. 9:**
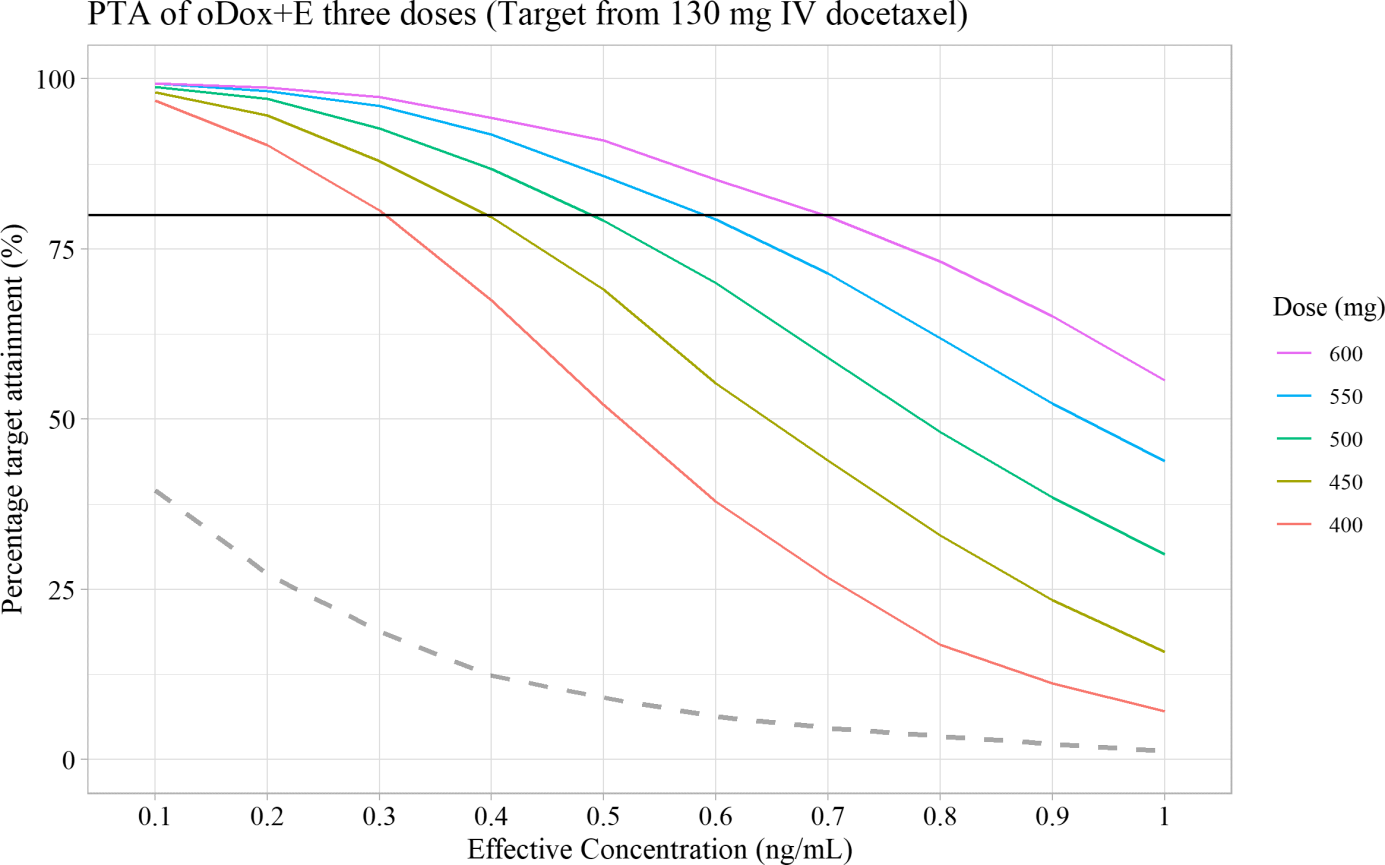
Probability target attainment of oDox + E dose between 400 to 600 mg given three times by dose level. Black line denotes 80% PTA. Dashed line denotes the PTA of a 600 mg dose of oral docetaxel alone (i.e., bioavailability of 8%) given three times. PTA – Probability target attainment

1000 simulations were performed for each oDox + E dose regimen and EC level combination outlined in Table 2. A total of 50 PTA values were calculated for each combination of oDox + E dose and EC included within the simulation space. These PTAs are shown in Figs. 7, 8 and 9f or a single dose, two doses and three doses of oDox + E, respectively.

The PTAs of a 600 mg dose of oral docetaxel alone (with a bioavailability of 8%) was added to each PTA figure as a dashed line. Oral docetaxel alone could not achieve a PTA > 80% for any dose regimen at any EC value.

#### GO / NO-GO framework

1. GO / NO-GO recommendation (i.e., existence of a practical regimen of oDox + E?)

No practical regimen of oDox + E existed with a single administration of oDox + E as none of the single dose regimens achieved a PTA of > 80% (Fig. 7). Practical regimens of oDox + E existed when two or three doses are administered (Figs. 8 and 9).

2. GO conditions

Scenario two of the GO conditions was observed for repeated dosing of oDox + E given either twice or three times, depending on the EC level. For the two-dose regimen of oDox + E (Fig. 8), the PTA > 80% was achieved across the 400-600 mg dose range at an EC of 0.1 ng/mL but could not be achieved for any dose regimen when the EC was greater than 0.5 ng/mL. For the three-dose regimen of oDox + E (Fig. 9), the PTA > 80% was achieved across the 400-600 mg dose range at an EC of less than 0.3 ng/mL but was only achieved by the 550 mg dose level and above when the EC was 0.5 ng/mL. The maximum dose regimen evaluated in this simulation (600 mg oDox + E given three times) could achieve a PTA > 80% when the EC was at or below 0.7 ng/mL. For an EC of 1.0 ng/mL, the maximum dose regimen evaluated produced a PTA of 56%, below the 80% power threshold.

Therefore, a GO recommendation with conditions was proposed for oDox + E. The recommendation prior to commencement of further clinical trials was to better quantify the EC value. If the EC were found to be less than 0.7 ng/mL there would be more confidence in a GO decision and progress to a Phase IIa/b trial, while an EC in excess of 1.0 ng/mL would strongly require consideration of a NO-GO decision and re-evaluation of the development strategy of oDox + E.

## Discussion

This study provides a real-world motivating example utilising a GO / NO-GO framework facilitated by MIDD at an early proof of concept phase. It aligns with FDA’s initiative to improve oncology drug development efficient via Project Optimus [3]. Whether a feasible regimen of oDox + E existed was the key question MIDD was deployed to answer using the data available at this early stage of development (i.e., prior to commencement of further studies such as bioequivalence, formulation, internal covariate, and external covariate studies). This approach demonstrates the potential of MIDD to improve drug development efficiency through clear action points depending on the answer to this question. In summary, a clear NO-GO would allow early termination and diversion of resources to alternative projects, a Conditional GO would guide the next studies to perform to address the most critical assumptions, finally, a clear GO would justify progression and investment of further resources to the development of oDox + E until the next GO / NO-GO point.

### Feasibility of oDox + E

The GO / NO-GO framework and simulations identified that a feasible regimen of oDox + E (relative to current IV docetaxel regimen) exists. Each simulated patient acted as their own “control” as their unique set of PK parameters were used to simulate the PK profiles for oDox + E and IV docetaxel. A bioequivalence margin of 20% [29] was applied when comparing the AUCOEC for oDox + E and IV docetaxel within a patient. A non-inferiority margin of 20% was applied to determine the threshold for the PTA. A single dose of oDox + E at 600 mg (the highest dose in the trial) produced a PTA of 74%, therefore, double, and triple dose regimens were explored. These regimens assumed that the patients would take a repeat dose 24 h after the initial dose and assumed the free concentration of docetaxel was reset to 0 (a potentially conservative assumption). The double and triple dose regimens were able to produce AUCOEC comparable to IV docetaxel. It should be noted that inference from the simulations was limited by the lack of toxicity or tolerability estimates for the multiple dosing regimen, which could not be included given the few side effects that occurred within the small patient cohort.

Interestingly, Aldaz et al. explored the role of the maximum plasma concentration (Cmax) and exposure of total docetaxel as AUC in early breast cancer patients receiving docetaxel 75-100 mg/m2. From the pharmacokinetic-pharmacodynamic analysis, the authors recommended a Cmax of < 3500 ng/mL and AUC > 4500 ng.h/mL target would minimise the incidence of adverse effects and increase the probability of efficacy [30]. These PK targets were derived from total plasma docetaxel concentration. In the context of this study, the importance of the Cmax for adverse effects and exposure for efficacy is highlighted. The use of AUC as the exposure metric is equivalent to the AUCOEC with an effective concentration of zero. Assuming the true effective concentration was closer to zero than a higher value we would expect the PTA [7–9] to be on the higher end giving more confidence that oDox + E would achieve the PTA target of > 80% when compared to IV docetaxel. Furthermore, if Cmax proves to be an important proxy for the adverse effects of docetaxel the oral route is likely to be much better tolerated as oDox + E attained a lower Cmax while achieving a not-inferior exposure when used in a multi-dose regimen.

### Modelling and simulation

A PK model was developed for IV docetaxel and oDox + E which included both total and unbound plasma concentration. Given the complexity of the four distinct groups (IV total, IV unbound, oDox + E total, and oDox + E unbound) within the dataset a stepwise approach was used to elucidate key model structure with the relevant amount of data required which had the advantage of minimising computational costs while retaining the structural information gained.

The bioavailability of oDox + E in the final model was estimated at 25% which is higher than the 8% bioavailability of oral docetaxel administered alone [10]. The three-fold increase reflects the effective inhibition of intestinal P-gp efflux pumps but does not overcome the extensive first pass metabolism that occurs hepatically. Importantly, the improvement in bioavailability was sufficient for oDox + E to achieve unbound PK exposures comparable to the current IV docetaxel regimen.

Time-varying binding structures were explored due to the possibility of excipient binding which may make the unbound fraction not constant with time. However, the exploration of these alternative binding models within the sub-models for IV docetaxel and oDox + E did result in an improvement in model fit. With a constant fraction unbound model, the final PK model estimates the fraction unbound after IV docetaxel and oDox + E administration to be 0.67% and 1.02% respectively. A discrepancy seems to exist between the two and we hypothesise that polysorbate 80 present in the IV formulation may contribute to the differential binding.

Due to the data set being limited to 9 patients, covariates were not included in the analysis. Furthermore, the small number of patients limited the ability to elucidate more nuanced model improvements when trialling different model features. Without the ability to make nuanced adjustments, model misspecification inevitably occurred such that potential non-linearity that may have occurred in some plasma concentration profiles beyond 12 h could not be accounted for (Supplement 4). However, these model-misspecifications are unlikely to have impacted the conclusions drawn from the simulation given the concentration range at which they occurred. Furthermore, the small patient cohort does not invalidate the GO / NO-GO decision framework that has been applied at this early proof of concept stage for oDox + E, where an opportunity to apply a GO / NO-GO decision point is provided based on the data available at this early stage. Future studies, as oDox + E progress, will result in more patient data becoming available to quantify the population PK parameters for oDox + E (i.e., from bioequivalence, formulation, and covariate studies as part of a proof-of-concept package).

The final 3-compartment PK model and parameters were comparable to previous published findings. Bruno et al. [31] found a 3-compartment model best described the PK of docetaxel and reported a total docetaxel CL and Vss of 36.7 L h^−1^ and 149L, respectively. This is comparable to our reported total docetaxel CL and Vss of 57.3 L h^−1^ and 425L, respectively. Despite the low number of patients included in the model the relative standard error for the parameters were mostly within an acceptable range of < 30%.

### Project optimus and dose optimisation

Analysis of the trial data using PK modelling, and application of a GO / NO-GO framework dependent on the performance of oDox + E compared to standard of care IV docetaxel regimen demonstrates the ability to improve the decision making and dose selection during drug development using a systematic approach. This is a focus for the Oncology Center of Excellence’s (OCE) Project Optimus initiative which aims to reform the dose optimisation and selection paradigm in oncology drug development to improve the efficiency of oncology drug development [2, 3]. For oDox + E, we used the current standard of care regimen as the baseline to compare the pharmacokinetic exposure to determine whether a feasible PK exposure could be achieved with a combination oral regimen. In answering this question, we identified an important gap in the knowledge and the need to further quantify “effective concentration” to reduce the risk of failure to progress development due to the absence of this knowledge. Furthermore, this uncertainty was able to be captured within the GO / NO-GO framework prior to performance of simulations.

## Conclusion

This study aligns with the FDA’s Project Optimus and demonstrates the value of MIDD at an early phase of oncology drug development using oDox + E as a motivating example. The key question answered by this study was whether a feasible regimen of oDox + E existed. The purpose of this question was to provide an early GO / NO-GO decision point to guide drug development and improve development efficiency. A population pharmacokinetic model was developed for the total and unbound concentration in plasma of docetaxel after administration of IV docetaxel and oDox + E. The model was used to simulate oDox + E dose regimens which were compared to the current standard of care IV docetaxel regimen. A GO / NO-GO framework was applied to determine whether oDox + E should progress to the next phase of drug development and whether any conditions should apply. A two or three-dose regimen of oDox + E at 600 mg was able to achieve non-inferior pharmacokinetic exposure to current standard of care IV docetaxel in simulations. A Conditional GO decision was made based on this result and further quantification of the “effective concentration” would improve the ability to optimise the dose regimen. This demonstrated a shift from the MTD paradigm to a more optimised and efficient approach for oncology drug development.

### Supplementary Information

Below is the link to the electronic supplementary material.

Supplementary file1 (DOCX 159 KB)

## References

[1] Takimoto CH (2009). Maximum tolerated dose: clinical endpoint for a bygone era?. Target Oncol.

[2] Murphy R, Halford S, Symeonides SN (2023). Project Optimus, an FDA initiative: Considerations for cancer drug development internationally, from an academic perspective. Front Oncol.

[3] FDA U. Project Optimus 2022 [Available from: https://www.fda.gov/about-fda/oncology-center-excellence/project-optimus.

[4] Fourie Zirkelbach J, Shah M, Vallejo J, Cheng J, Ayyoub A, Liu J (2022). Improving dose-optimization processes used in oncology drug development to minimize toxicity and maximize benefit to patients. J Clin Oncol.

[5] Sparano JA (2000). Taxanes for breast cancer: an evidence-based review of randomized phase II and phase III trials. Clin Breast Cancer.

[6] Schrijvers D, Vermorken JB (2005). Taxanes in head and neck cancer. Future Oncol.

[7] Van Cutsem E (2004). The treatment of advanced gastric cancer: new findings on the activity of the taxanes. Oncologist.

[8] Tannock IF, de Wit R, Berry WR, Horti J, Pluzanska A, Chi KN (2004). Docetaxel plus prednisone or mitoxantrone plus prednisone for advanced prostate cancer. N Engl J Med.

[9] Reck M, Kaiser R, Mellemgaard A, Douillard J-Y, Orlov S, Krzakowski M (2014). Docetaxel plus nintedanib versus docetaxel plus placebo in patients with previously treated non-small-cell lung cancer (LUME-Lung 1): a phase 3, double-blind, randomised controlled trial. Lancet Oncol.

[10] Malingre MM, Richel DJ, Beijnen JH, Rosing H, Koopman FJ, Ten Bokkel Huinink WW (2001). Coadministration of cyclosporine strongly enhances the oral bioavailability of docetaxel. J Clin Oncol.

[11] Oostendorp RL, Huitema A, Rosing H, Jansen RS, Ter Heine R, Keessen M (2009). Coadministration of ritonavir strongly enhances the apparent oral bioavailability of docetaxel in patients with solid tumors. Clin Cancer Res.

[12] Schwartzberg LS, Navari RM (2018). Safety of Polysorbate 80 in the Oncology Setting. Adv Ther.

[13] Loos WJ, Baker SD, Verweij J, Boonstra JG, Sparreboom A (2003). Clinical pharmacokinetics of unbound docetaxel: role of polysorbate 80 and serum proteins. Clin Pharmacol Ther.

[14] Minami H, Kawada K, Sasaki Y, Igarashi T, Saeki T, Tahara M (2006). Pharmacokinetics and pharmacodynamics of protein-unbound docetaxel in cancer patients. Cancer Sci.

[15] Liu G, Franssen E, Fitch MI, Warner E (1997). Patient preferences for oral versus intravenous palliative chemotherapy. J Clin Oncol.

[16] Sohi GK, Levy J, Delibasic V, Davis L, Mahar A, Amirazodi E (2020). The cost of chemotherapy administration: A systematic review and meta-analysis. Am Soc Clin Oncol.

[17] Kim TE, Gu N, Yoon SH, Cho JY, Park KM, Shin SG (2012). Tolerability and pharmacokinetics of a new P-glycoprotein inhibitor, HM30181, in healthy Korean male volunteers: single- and multiple-dose randomized, placebo-controlled studies. Clin Ther.

[18] Umanzor Funez GA, Vassallo RH, Chivalan Castro MA, Bejarano SA, Ramirez Velasquez JR, Kowalyszyn R, et al (2019) KX-ORAX-001: An open label, randomized, multicenter, phase III registrational study to determine the safety, tolerability, and tumor response of oraxol (HM30181A+ oral paclitaxel) and its comparability to IV paclitaxel in patients with metastatic breast cancer (MBC). Am Soc Clin Oncol

[19] Bruno R, Hille D, Riva A, Vivier N, ten Bokkel Huinnink WW, van Oosterom AT (1998). Population pharmacokinetics/pharmacodynamics of docetaxel in phase II studies in patients with cancer. J Clin Oncol.

[20] Gustafson DL, Long ME, Zirrolli JA, Duncan MW, Holden SN, Pierson AS (2003). Analysis of docetaxel pharmacokinetics in humans with the inclusion of later sampling time-points afforded by the use of a sensitive tandem LCMS assay. Cancer Chemother Pharmacol.

[21] Jackson C, Ou Y-C, Chao T-Y, En M, Hung NA, Wang D, et al. An open-label, pharmacokinetic study to determine the bioavailability, safety and tolerability of single dose oral docetaxel (Oradoxel) in metastatic prostate cancer (mPC) patients treated with IV docetaxel.

[22] Posdzich P, Darr C, Hilser T, Wahl M, Herrmann K, Hadaschik B (2023). Metastatic Prostate Cancer-A Review of Current Treatment Options and Promising New Approaches. Cancers (Basel).

[23] Wang D, Hung T, Hung N, Glue P, Jackson C, Duffull S (2023) Optimal sample selection applied to information rich, dense data. J Pharmacokinet Pharmacodyn 10.1007/s10928-023-09883-7 37561265

[24] Irby DJ, Ibrahim ME, Dauki AM, Badawi MA, Illamola SM, Chen M (2021). Approaches to handling missing or “problematic” pharmacology data: Pharmacokinetics. CPT: Pharmacometrics Syst Pharmacol.

[25] Koo AN, Min KH, Lee HJ, Lee SU, Kim K, Kwon IC (2012). Tumor accumulation and antitumor efficacy of docetaxel-loaded core-shell-corona micelles with shell-specific redox-responsive cross-links. Biomaterials.

[26] Bissery MC, Vrignaud P, Lavelle F (1995). Preclinical profile of docetaxel (taxotere): efficacy as a single agent and in combination. Semin Oncol.

[27] Lavelle F, Bissery MC, Combeau C, Riou JF, Vrignaud P, Andre S (1995). Preclinical evaluation of docetaxel (Taxotere). Semin Oncol.

[28] Bioequivalence RA (1992). Pharm Res.

[29] Carpenter D, Tobbell DA (2011) Bioequivalence: the regulatory career of a pharmaceutical concept. Bull Hist Med 93–131 10.1353/bhm.2011.0024 21551918

[30] Aldaz A, Schaiquevich P, Aramendía JM (2023). A pharmacometrics model to define docetaxel target in early breast cancer. Br J Clin Pharmacol.

[31] Bruno R, Vivier N, Vergniol JC, De Phillips SL, Montay G, Sheiner LB (1996). A population pharmacokinetic model for docetaxel (Taxotere): model building and validation. J Pharmacokinet Biopharm.

