# Correction: Oral docetaxel plus encequidar – a pharmacokinetic model and evaluation against IV docetaxel

**DOI:** 10.1007/s10928-024-09924-9

**Published:** 2024-05-14

**Authors:** David Wang, Chris Jackson, Noelyn Hung, Tak Hung, Rudolf Kwan, Wing-Kai Chan, Albert Qin, Natalie J. Hughes-Medlicott, Paul Glue, Stephen Duffull

**Affiliations:** 1 Department of Anaesthesia, Waikato Hospital, Hamilton, New Zealand; 2 Department of Medicine, University of Otago, Dunedin, New Zealand; 3 Department of Pathology, University of Otago, Dunedin, New Zealand; 4 Zenith Technology Limited, Dunedin, New Zealand; 5 Athenex Inc, New York, USA; 6 PharmaEssentia Corporation, Taipei, Taiwan; 7 School of Pharmacy, University of Otago, Dunedin, New Zealand; 8 Department of Psychological Medicine, University of Otago, Dunedin, New Zealand; 9 Certara, Princeton, NJ USA

**Correction to: Journal of Pharmacokinetics and Pharmacodynamics**

10.1007/s10928-024-09913-y

In this article the author affiliation information for Wing Kai Chan was inaccurate. The affiliation was listed as “PharmaEssentia Corporation, Taipei, Taiwan,” but it should be “Athenex Inc, New York, USA.”

A discrepancy in the sequence of authors and respective affiliation for authors Rudolf Kwan, Wing Kai Chan, Albert Qin, Natalie J. Hughes Medlicott, Paul Glue and Stephen Duffull has been corrected.

The original article has been corrected.

