# Supplementary material for: Oral docetaxel plus encequidar – A pharmacokinetic model and evaluation against IV docetaxel
Source: J Pharmacokinet Pharmacodyn. 2024 Mar 19;51(4):335–52. doi: 10.1007/s10928-024-09913-y (PMC11254990; doi:10.1007/s10928-024-09913-y)
Supplement: Supplementary file 1 — Supplementary file1 (DOCX 159 KB) [file 10928_2024_9913_MOESM1_ESM.docx]

**Supplements**

*Supplement 1*

|  |  | **Final Model**  **(Combined)** | **oDox+E** **Model** | **IV Docetaxel Model** |
| --- | --- | --- | --- | --- |
| **Sub-model** |  | **6** | **5** | **4** |
| **Data** |  | **All** | **PO Total \|**  **PO Unbound** | **IV Total \|**  **IV Unbound** |
| **Compartments** |  | **3** | **2** | **3** |
|  |  |  |  |  |
| **Parameter** | **Units** |  |  |  |
| CL(/F) | L h^-1^ | 8570 (15%) | 35800 (24%) * | 7610 (17%) |
| F |  | 25% (30%) | - | - |
| ka | h^-1^ | 0.223 (19%) | 0.898 (51%) * | - |
| f_unbound IV_ | % | 0.67% (18%) |  | 0.74% (16%) |
| f_unbound PO_ | % | 1.02% (26%) | 0.99% (25%) |  |
| ALAG | h | 0.242 (1%) | 0.25 (<1%) * | - |
| Vc | L | 3500 (24%) | 128000 (38%) * | 3020 (42%) |
| Qp1 | L h^-1^ | 1250 (25%) | 20800 (13%) * | 1160 (33%) |
| Vp1 | L | 9450 (69%) | 512000 (22%) * | 10200 (96%) |
| Qp2 | L h^-1^ | 2810 (14%) | - | 2270 (16%) |
| Vp2 | L | 61900 (15%) | - | 49100 (18%) |
|  |  |  |  |  |
| **BSV (CV%)** |  |  |  |  |
| ω_CL_ |  | 27% (64%) | 57% (75%) | 21% (84%) |
| ω_logitF_ |  | 0.71 (51%) ** | - | - |
| ωk_a_ |  | 47% (60%) | 135% (60%) | - |
| ω_ALAG_ |  | 3% (110%) | 68% (51%) | - |
| ω_Vc_ |  | 63% (51%) | 90% (71%) | 55% (52%) |
| ω_Vp1_ |  | 148% (48%) | 31% (99%) | 155% (71%) |
| ω_Vp2_ |  | 0% (NA) | - | 0% (0%) |
| ω_BIV_ |  | 28% (62%) | - | 19% (81%) |
| ω_BPO_ |  | 72% (48%) | 48% (68%) | - |
|  |  |  |  |  |
| Tot IV σ_add_ | ng mL^-1^ | <0.001 (NA) | - | <0.001 (NA) |
| Tot IV σ_prop_ |  | 0.143 (12%) | - | 0.137 (12%) |
|  |  |  |  |  |
| UF IV σ_add_ | ng mL^-1^ | 0.005 (139%) | - | 0.0003 (607%) |
| UF IV σ_prop_ |  | 0.538 (36%) | - | 0.633 (32%) |
|  |  |  |  |  |
| Tot PO σ_add_ | ng mL^-1^ | 0.199 (181%) | 0.0001 (1%) | - |
| Tot PO σ_prop_ |  | 0.322 (14%) | 0.173 (14%) | - |
|  |  |  |  |  |
| UF PO σ_add_ | ng mL^-1^ | 0.003 (99%) | 0.0004 (829%) | - |
| UF PO σ_prop_ |  | 0.337 (18%) | 0.406 (14%) | - |

**Pharmacokinetic model parameters for final model (combined) and models describing oDox+E** **and IV Docetaxel individually.** ***Parameter values for the oDox+E model are apparent parameters with regards to bioavailability (F), that is Parameter/F. The relative standard errors (RSE) are shown in brackets next to the parameter values**.** ω values are shown as coefficient of variation percentages (CV) calculated as the square root of the ω value.

*Abbreviations: PO Total | PO Unbound – Dataset of total and unbound concentration of docetaxel in plasma over time after oDox+E administration; IV Total | IV Unbound* *– Dataset of total and unbound concentration of docetaxel in plasma over time after IV administration of docetaxel; CL(/F) – Clearance or apparent clearance; F – Bioavailability; KA – Absorption rate constant; f_unbound IV_ – Fraction unbound for IV docetaxel, equivalent to the inverse of IV Docetaxel Binding constant; f_unbound PO_ – Fraction unbound for oDox+E , equivalent to the inverse of oDox+E Binding constant ; ALAG – Lag time; Vc – Central compartment apparent volume; Qp1 – Intercompartmental clearance between central compartment and first peripheral compartment; Vp1 – First peripheral compartment apparent volume; Qp2 – Intercompartmental clearance between central compartment and second peripheral compartment; Vp2 – Second peripheral compartment apparent volume; BSV – Between subject variability; ω_CL_ – BSV of CL; ω_logitF_ – BSV of logitF; ω_ka_ – BSV of ka; ω_ALAG_ – BSV of ALAG; ω_Vc_ – BSV of Vc; ω_Vp1_ – BSV of Vp1; ω_Vp2_ – BSV of Vp2; ω_BIV_ – BSV of BIV; ω_BPO_ – BSV of BPO; Tot IV σ_add_ – Additive residual error for total concentration of docetaxel after IV administration; Tot IV σ_prop_ – Proportional residual error for total concentration of docetaxel after IV administration (Fraction); UF IV σ_add_ – Additive residual error for unbound concentration of docetaxel after IV administration; UF IV σ_prop_ – Proportional residual error for unbound concentration of docetaxel after IV administration (Fraction); Tot PO σ_add_ – Additive residual error for total concentration of docetaxel after oDox+E administration; Tot PO σ_prop_ – Proportional residual error for total concentration of docetaxel after oDox+E administration (Fraction); UF PO σ_add_ – Additive residual error for unbound concentration of docetaxel after oDox+E administration; UF PO σ_prop_ – Proportional residual error for unbound concentration of docetaxel after oDox+E administration (Fraction).*

*Supplement 2*


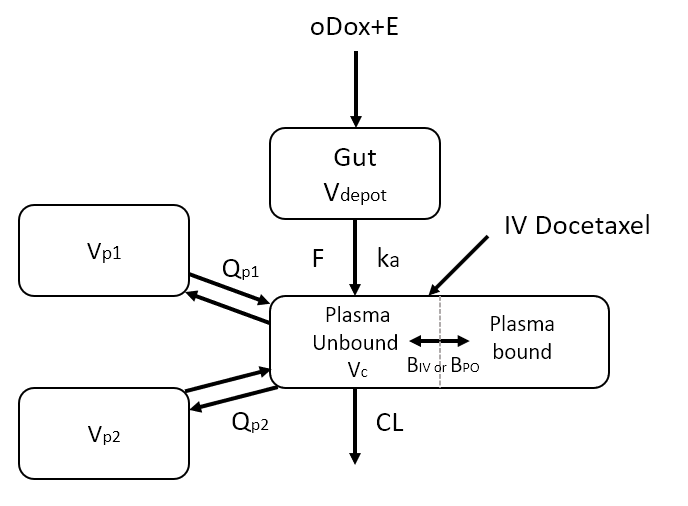


**Structure of final model for docetaxel.**

*Abbreviations: oDox+E – Oral Docetaxel with Encequidar;* *V_depot_ – Volume of depot compartment; V_c_ – Volume of central compartment / plasma; V_p1_ – Volume of first peripheral compartment; V_p2_ – Volume of second peripheral compartment; F – Bioavailability; ka – Absorption rate constant; Q_p1_ – Intercompartmental clearance between central compartment and first peripheral compartment; Q_p2_ – Intercompartmental clearance between central compartment and second peripheral compartment; CL – clearance; B_IV_ – Binding constant for IV docetaxel, equivalent to 1 / Fraction unbound; B_PO_ – Binding constant for oDox+E, equivalent to 1 / Fraction unbound.*

*Supplement 3*

| A  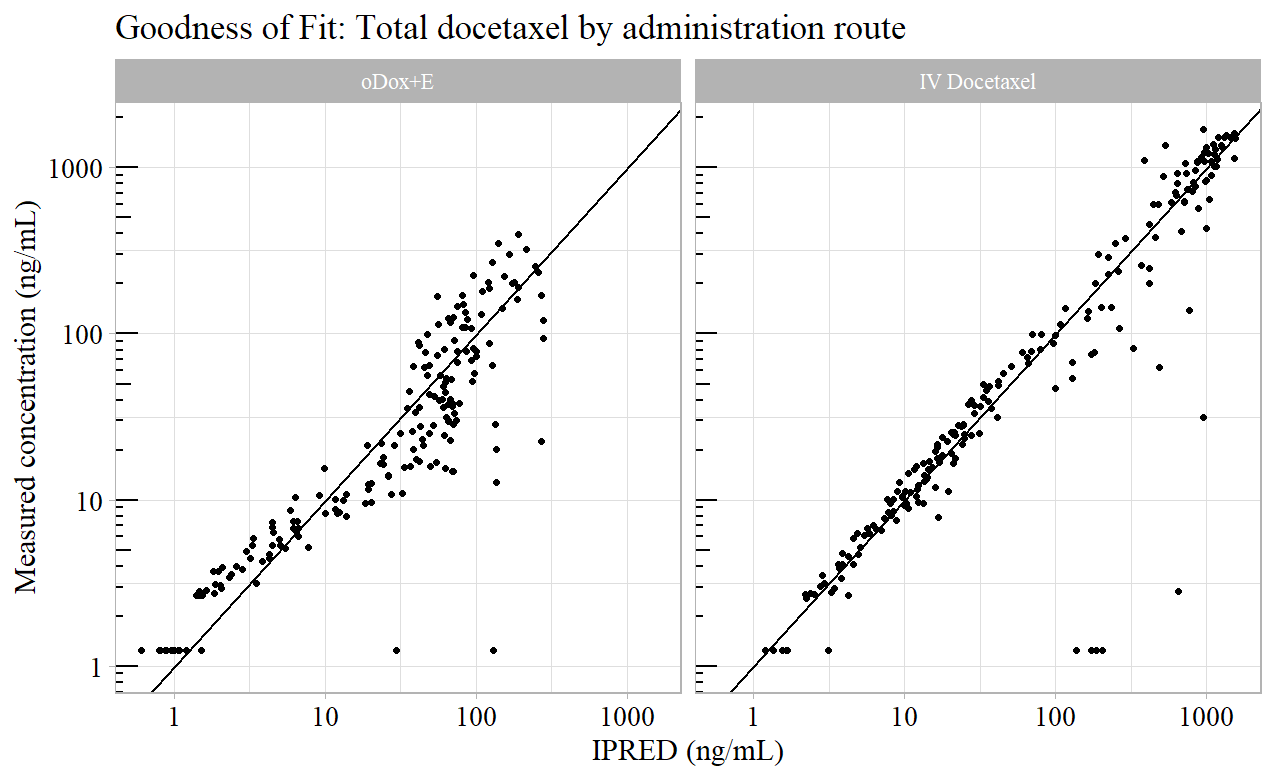 |
| --- |
| B  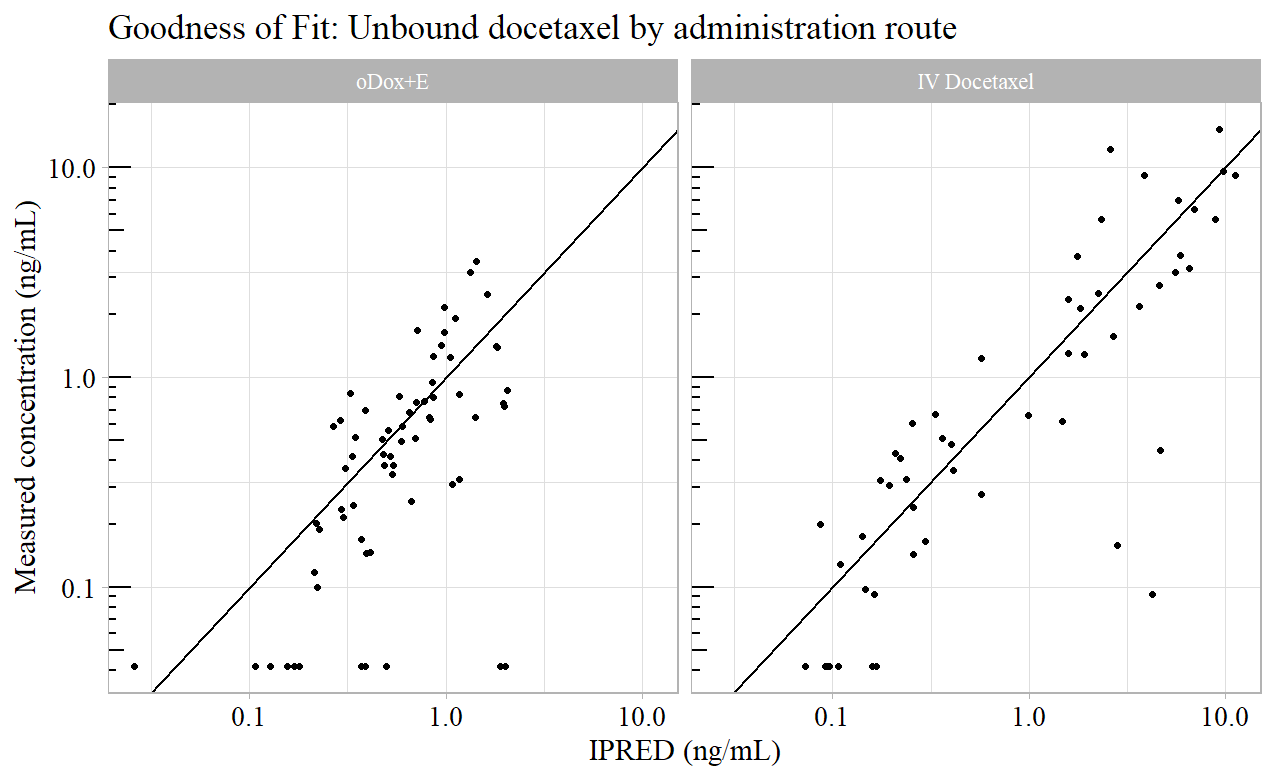 |
| **Goodness of fit plots**. Top figure (A) shows the goodness of fit for the **total** docetaxel concentrations predictions against the actual measured concentrations by administration route. The bottom figure (B) shows the goodness of fit for the **unbound** docetaxel predictions from the model against the actual measured concentrations by administration route.  *Supplement 4*  **Model misspecification examples**  Non-linearity was noted in some individual profiles and explored during the model building process. Simulation was also used to determine whether profiles could be recreated using different model structures as described in the main text.  **oDox+E example**: Potential non-linearity was seen in 24-80 hour range of some oDox+E profiles where there is an increase in the total concentration of docetaxel creating a second peak. This is shown around the 30h mark in **Figure A** below. Possible mechanisms for this phenomena include 1) non-linearity in disposition with a dual linear and non-linear process (this was explored in the model above but did not make a significant improvement), 2) dual absorption rates from enterohepatic cycling, formulation related effects or variable gastric motility and 3) time varying bioavailability or binding (also explored in the model above but did not make a significant improvement). Overall, the model misspecification would result in an under-estimation of the time over effective concentration for oDox+E when compared to IV docetaxel effectively, producing a more conservative oDox+E vs IV docetaxel exposure comparison for GO / NO-GO decision making.   \| 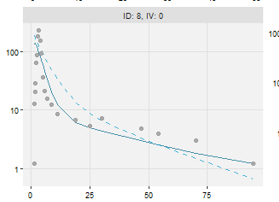 \| 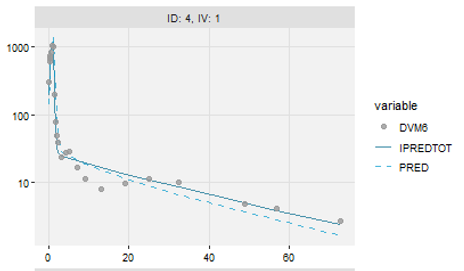 \| \| --- \| --- \| \| **Figure A** – Non-linearity and a “second peak” after oDox+E occurring around the 30 hour mark. \| **Figure B** – Non-linearity and a “triple peak” after IV docetaxel administration \| |

**IV docetaxel example:** A triple peak was seen in an IV docetaxel PK profile for one patient shown in **Figure B**. This phenomenon was not observed in any other profiles. This was not able to be described adequately. Overall, the model misspecification would result in an over-estimation of the time over effective concentration for IV docetaxel again producing a more conservative oDox+E vs IV docetaxel exposure comparison for GO / NO-GO decision making.
